# Supplementary material for: Wavelength engineerable porous organic polymer photosensitizers with protonation triggered ROS generation
Source: Nat Commun. 2023 Mar 17;14:1498. doi: 10.1038/s41467-023-37156-x (PMC10023675; doi:10.1038/s41467-023-37156-x)
Supplement: Supplementary file 1 — Supplementary Information [file 41467_2023_37156_MOESM1_ESM.pdf]

Supplementary Information

Wavelength engineerable porous organic polymer photosensitizers with protonation triggered ROS generation

Jinwoo Shin[1,2,†], Dong Won Kang[1,†], Jong Hyeon Lim[3,†], Jong Min An[4,†], Youngseo Kim[1], Ji Hyeon Kim[1], Myung Sun Ji[1], Sungnam Park[1,*], Dokyoung Kim[4,5,6,7,*], Jin Yong Lee[3,*], Jong Seung Kim[1,*], and Chang Seop Hong[1,*]

[1]Department of Chemistry, Korea University, Seoul 02841, Republic of Korea.

[2]Department of Chemistry, Sarafan ChEM-H Institute, and Stanford Cancer Institute, Stanford University, Stanford, CA, 94305, USA.

[3]Department of Chemistry, Sungkyunkwan University, Suwon 16419, Republic of Korea.

[4]Department of Biomedical Science, Graduate School, Kyung Hee University, Seoul 02447, Republic of Korea.

[5]Department of Anatomy and Neurobiology, College of Medicine, Kyung Hee University, Seoul 02447, Republic of Korea.

[6]KHU-KIST Department of Converging Science and Technology, Kyung Hee University, Seoul 02447, Republic of Korea.

[7]UC San Diego Materials Research Science and Engineering Center, 9500 Gilman Drive, La Jolla, CA 92093, USA.

[†]These authors contributed equally to this work.

*Corresponding authors: spark8@korea.ac.kr (S.P.), dkim@khu.ac.kr (D.K.), jinylee@skku.edu (J.Y.L.), jongskim@korea.ac.kr (J.S.K.), cshong@korea.ac.kr (C.S.H.)

Table of Contents

Section I. Physical measurements 3

Section II. Characterization data of porous organic photosensitizers 4

Supplementary Figs. 1–14

Section III. ROS generation ability of porous organic photosensitizers.......... 12

Supplementary Figs. 15–24

Section IV. Mechanism-related studies of porous organic photosensitizers ... 18

Supplementary Figs. 25–28

Supplementary Tables 1–2

Section V. Biological data of porous organic photosensitizers......................... 21

Supplementary Fig. 29–32

Section I. Physical measurements

Deionized water in the experiment was purified by aqua MAX^{TM} Basic360 series. Powder X-ray diffraction patterns were recorded using Cu Kα (λ = 1.5406 Å) on a Rigaku Ultima III diffractometer with a scan speed of 2°/min and a step size of 0.01°. Infrared spectra were obtained using a Nicolet iS10 FT-IR spectrometer with an ATR module. XPS data were measured at Semiconductor & Display Green Manufacturing Research Center at Korea University using X-tool. Elemental analysis measurements for C, H, and N were performed at the Elemental Analysis Service Center of Sogang University. Solid state NMR experiments are acquired from Bruker AVANCE II$^+$ 400MHz NMR system at KBSI Seoul Western Center. Thermogravimetric analysis (TGA) was carried out in N_2 (99.999 %) atmosphere (flow rate = 30 mL min^{-1}) in the temperature range 30 – 900 ºC (heating rate = 10 ºC min^{-1}) using a Scinco TGA-N 1500. Gas sorption measurements with N_2 (99.999%) and CO_2 (99.999%) were executed on a Micromeritics ASAP2020 instrument with up to 1 atm of gas pressure. SEM images were obtained from KBSI Seoul Center using Hitachi SU-70. Transmission Electron Microscopy (TEM) was carried out using a Tecnai G2 20 S-Twin microscope. The contact angle was measured by Phoenix-MT(T). Zeta-potential was measured using Malvern Instruments Zetasizer Nano ZS90 (Worcester-shire, UK). Solid-state UV-Vis data were collected at KBSI Daegu Center using Optical Spectrometer (UV/VIS/NIR spectrophotometer). UV-Vis absorbance spectra were obtained using Jasco V-750. Dissolved oxygen level was measured using a portable dissolved oxygen meter GHI 9147 under a gas-sealed environment to prevent oxygen leakage. EPR measurements were executed at KBSI Seoul Western Center and Kangwon National University using CW/Pulse EPR System with the condition (frequency: 9.64, power: 3 mW, modulation frequency: 100 kHz, modulation amplitude: 10 G, sweep time: 48 s, temperature: RT). Cyclic voltammetry data were obtained using a potentiostat (eDAQ, EA161).

Section II. Characterization data of porous organic photosensitizers

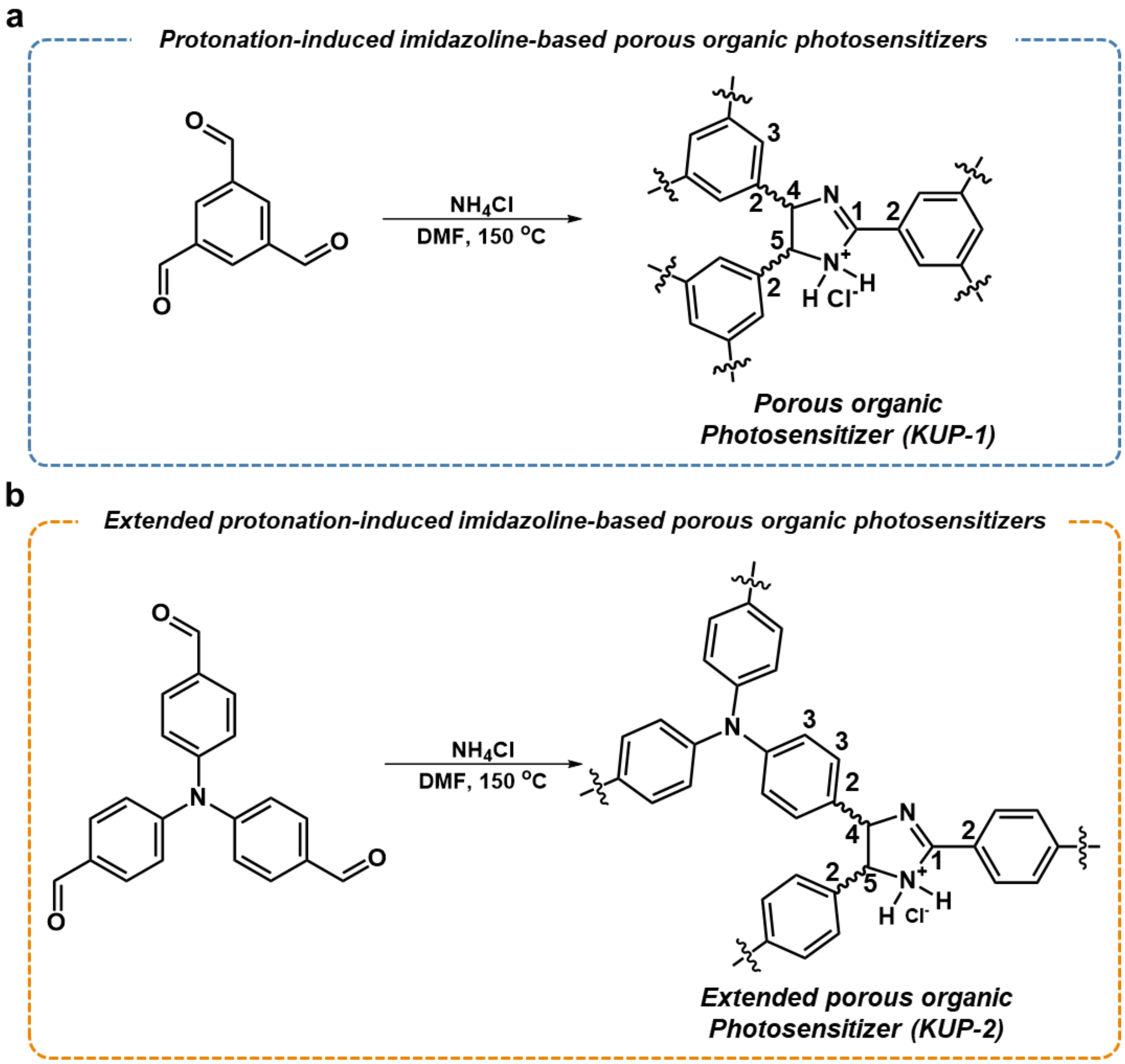

Supplementary Fig. 1 Synthetic schemes for **a KUP-1** and **b KUP-2**. KUP system was synthesized from cost-effective starting materials through a one-pot reaction.

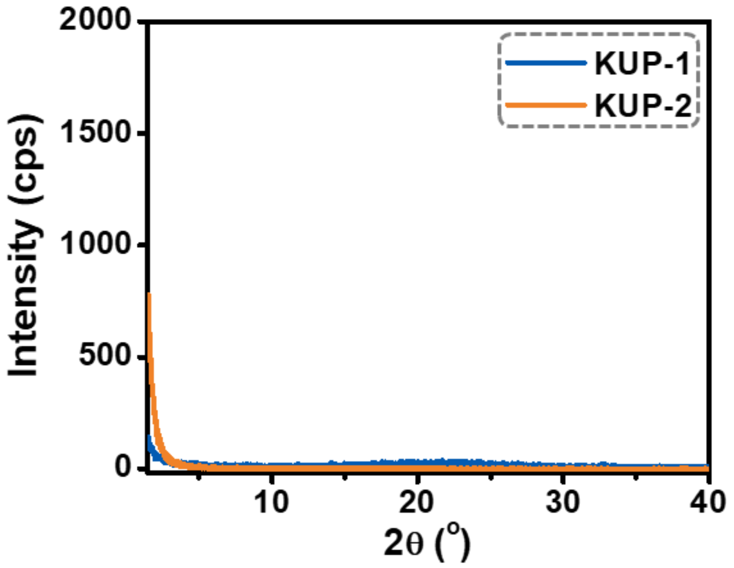

Supplementary Fig. 2 Power X-ray diffraction patterns of POPs. No distinct peaks were founded in the patterns, indicating amorphous characteristics of the porous polymers.

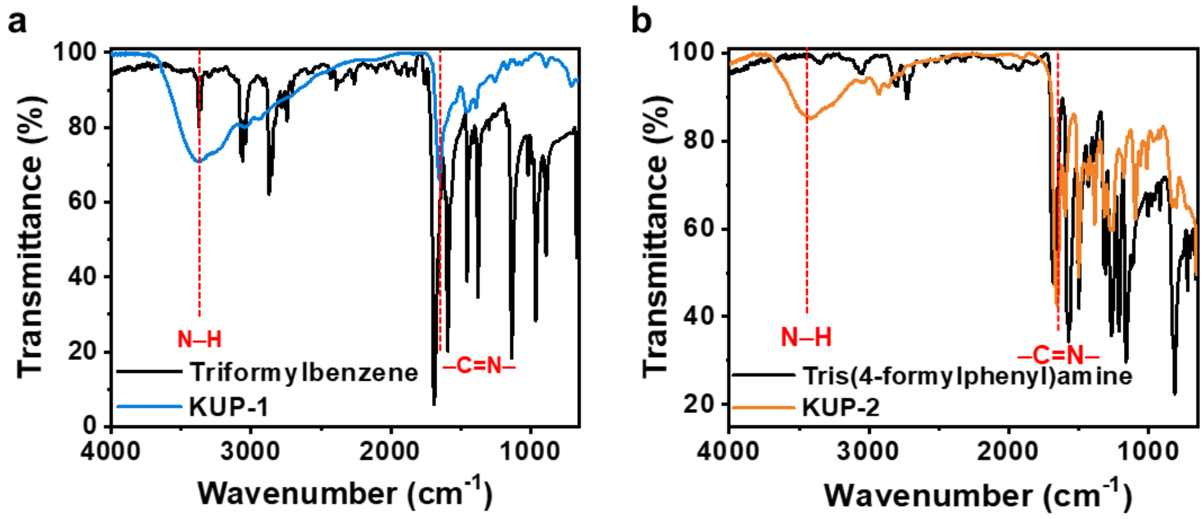

Supplementary Fig. 3 IR data of starting materials and porous organic polymers (**a** triformylbenzene and **KUP-1**, **b** tris(4-formylphenyl)amine and **KUP-2**). The new peaks were mainly related to the formation of protonated imidazoline moiety in the frameworks.

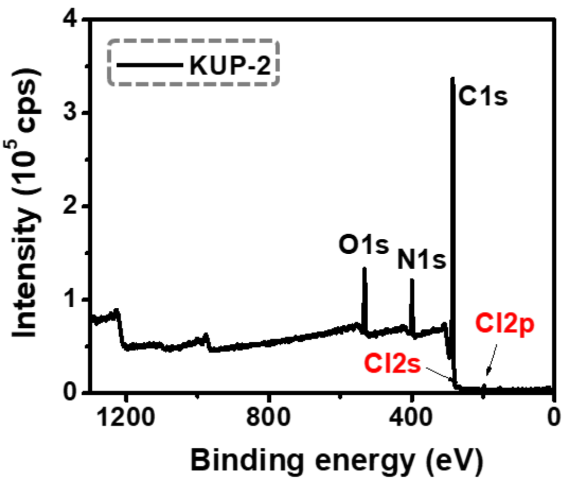

Supplementary Fig. 4 XPS survey scan data of **KUP-2**. Distinct Cl2s and Cl2p orbital peaks were observed in the spectrum. We expect that the role of chlorine is a counter anion of protonated imidazoline.

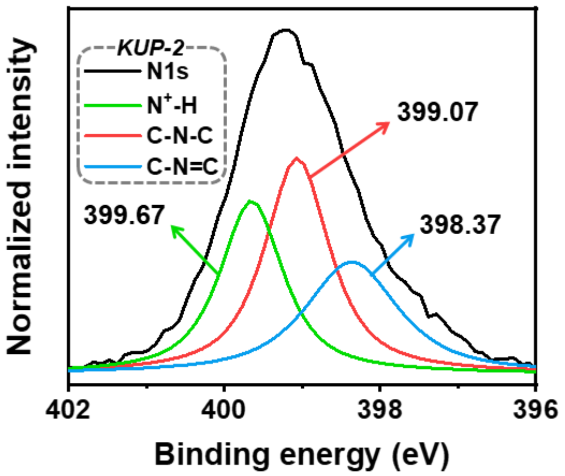

Supplementary Fig. 5 XPS narrow scan of N1s peak of **KUP-2**. **KUP-2** has various chemical environments of nitrogens in the framework.

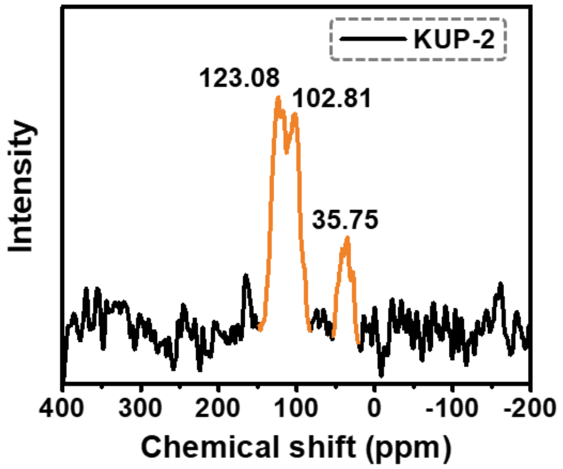

Supplementary Fig. 6 Solid-state ^{15}N NMR data of **KUP-2** (^{15}N CP/MAS - spinning rate: 6 kHz, delay time(d1): 3 s, contact time (p15): 2 ms, radio frequency: 40.54 MHz, calibration: NH_3NO_2 neat).

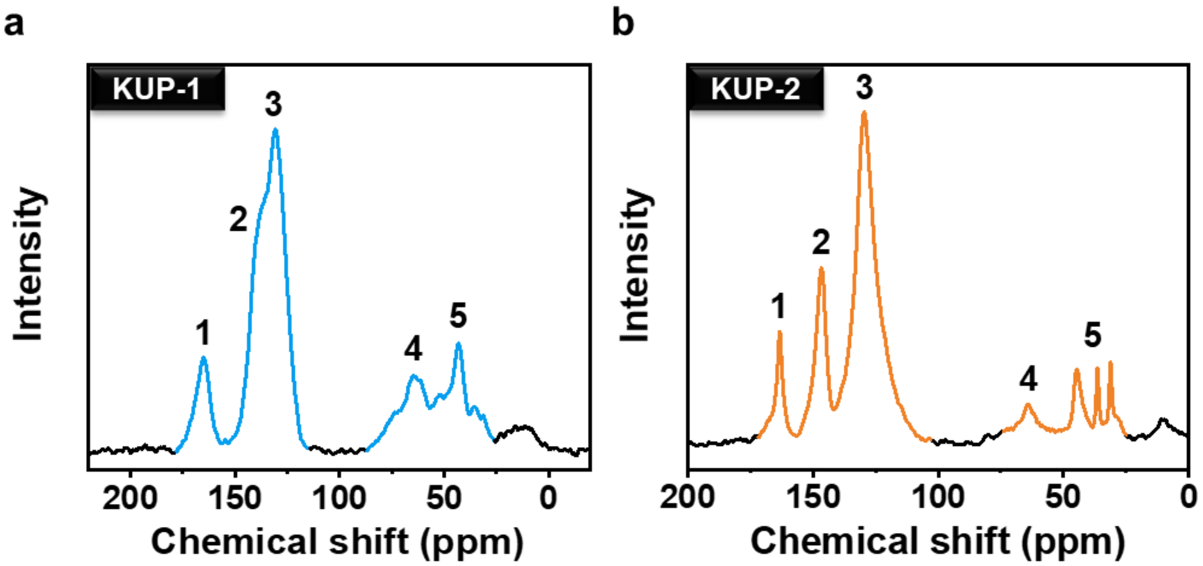

Supplementary Fig. 7 Solid-state ^{13}C NMR data of **a KUP-1** and **b KUP-2** (^{13}C CP/MAS - spinning rate: 12 kHz, delay time (d1): 3 s, contact time (p15): 2 ms, radio frequency: 100.4 MHz, calibration: TMS 0 ppm). Symbol (*) indicates unreacted aldehyde groups.

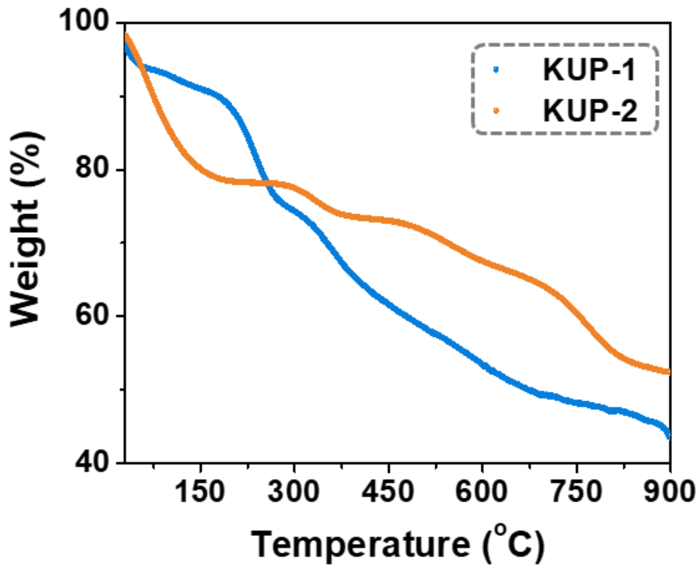

Supplementary Fig. 8 Thermogravimetric analysis profile of POPs. The initial weight loss is caused by the loss of guest molecules, such as water, from the pores of the framework.

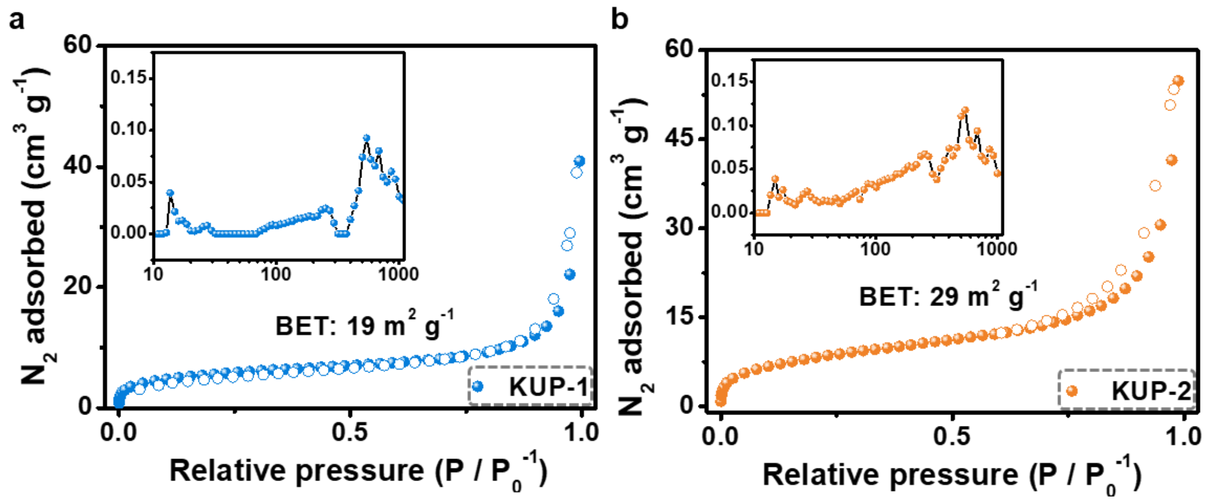

Supplementary Fig. 9 N_2 isotherms at 77 K and pore size distributions of **a KUP-1** and **b KUP-2**. The horizontal and vertical axes in the insets of panels represent pore width (Å) and differential pore volume (cm^3 g^{-1}).

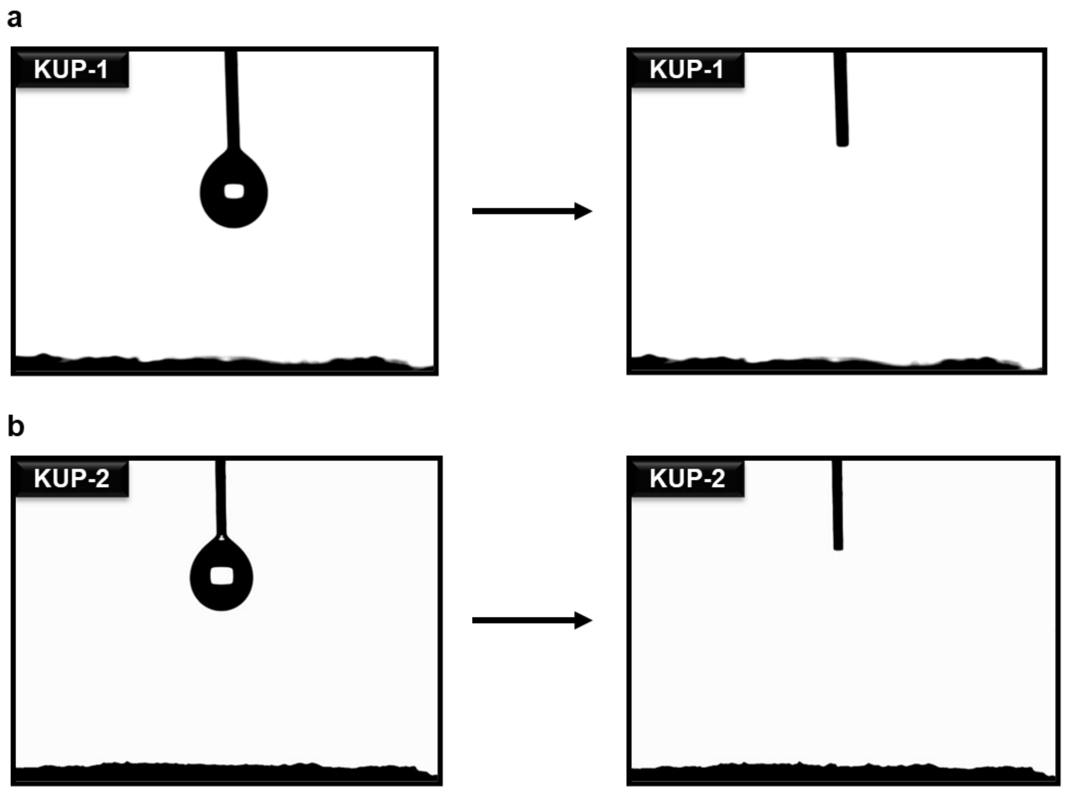

Supplementary Fig. 10 Water droplet test images of **a KUP-1** and **b KUP-2**. No water contact angle occurred due to the hydrophilicity of POPs.

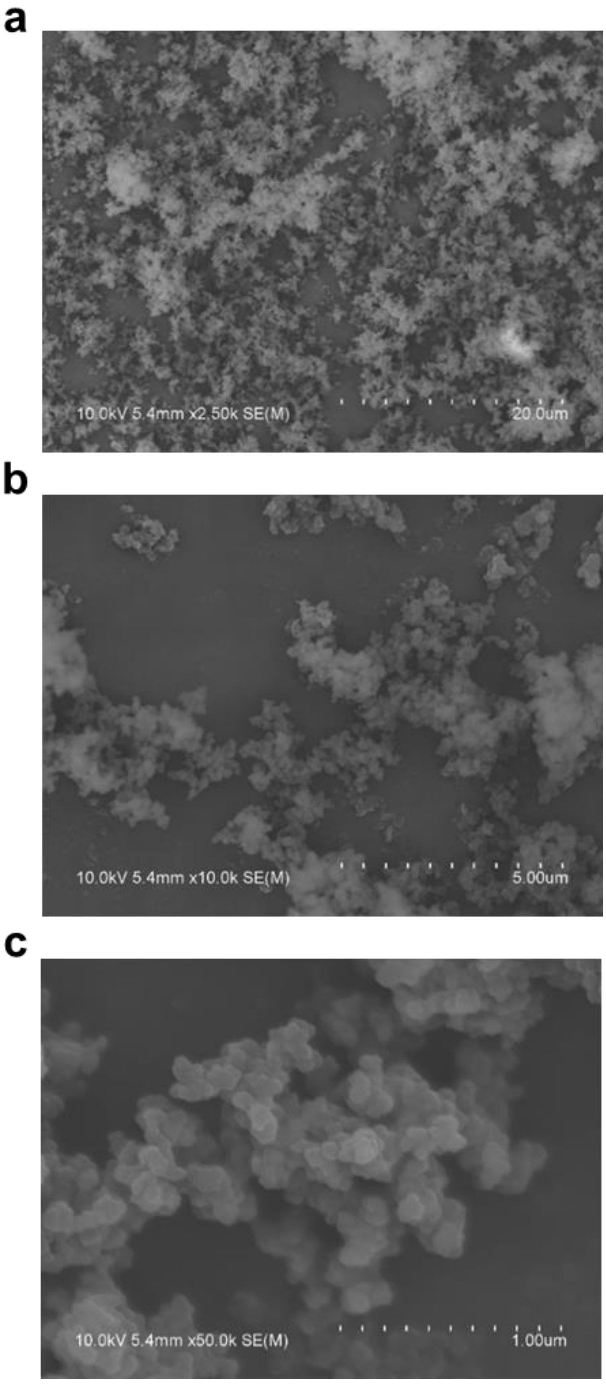

Supplementary Fig. 11 SEM images of **KUP-1** (**a**: x 2,500, **b**: x 10,000, **c**: x 50,000).

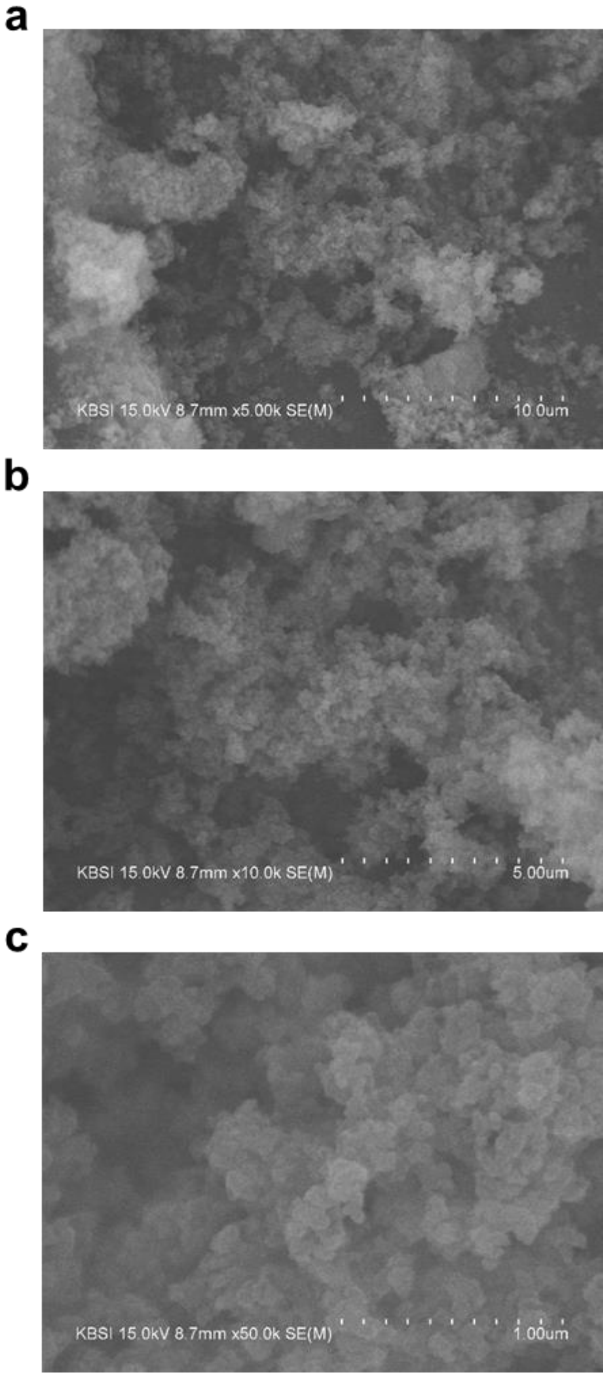

Supplementary Fig. 12 SEM images of **KUP-2** (**a**: x 5,000, **b**: x 10,000, **c**: x 50,000).

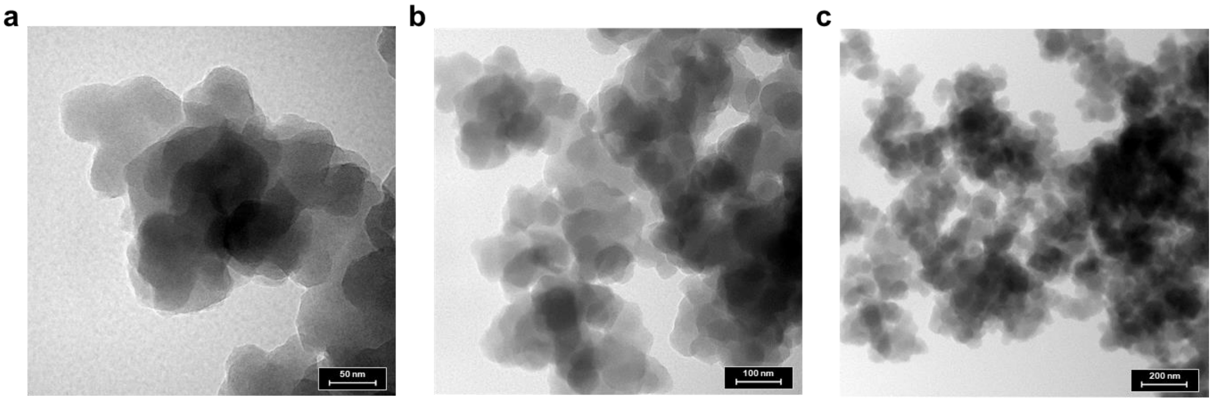

Supplementary Fig. 13 TEM images of **KUP-1** (**a**: x 250,000, **b**: x 125,000, **c**: x 63,000).

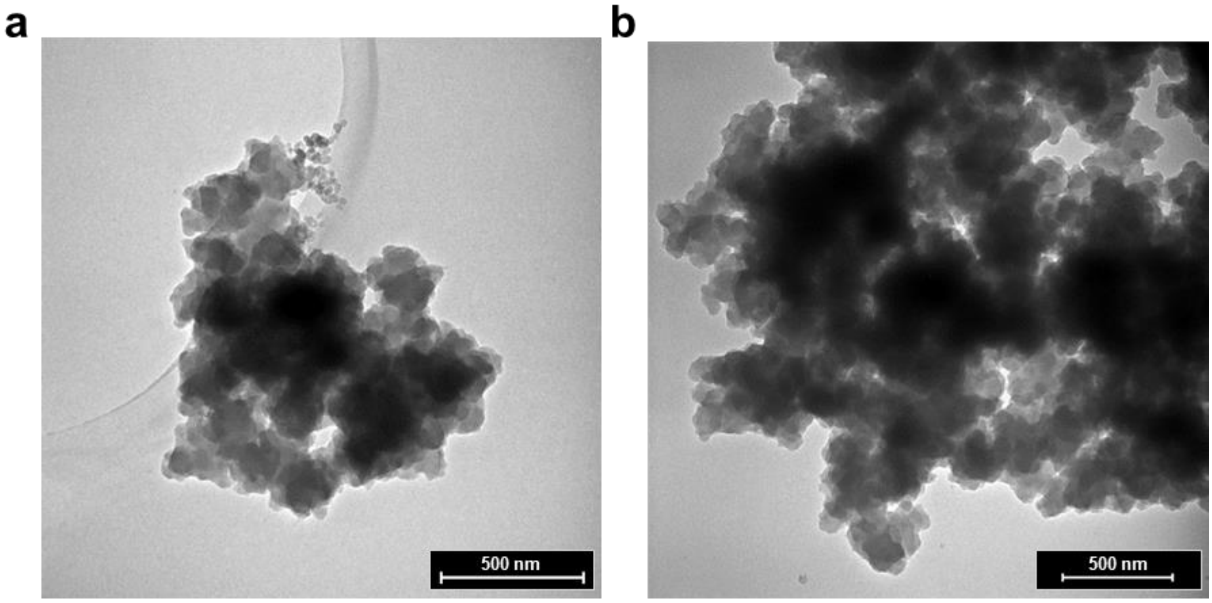

Supplementary Fig. 14 TEM images of **KUP-2** (**a**: x 43,000, **b**: x 43,000).

Section III. ROS generation ability of porous organic photosensitizers

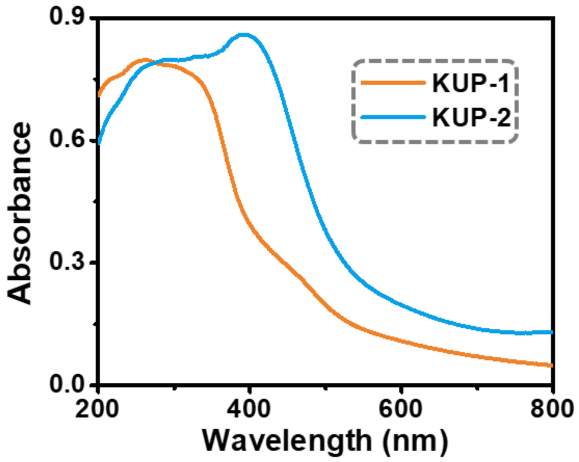

Supplementary Fig. 15 A solid-state UV-Vis spectrum of POPs. The strong absorbance bands of POPs were observed overall visible range, but a maximum absorbance peak was shifted to red in the extended system.

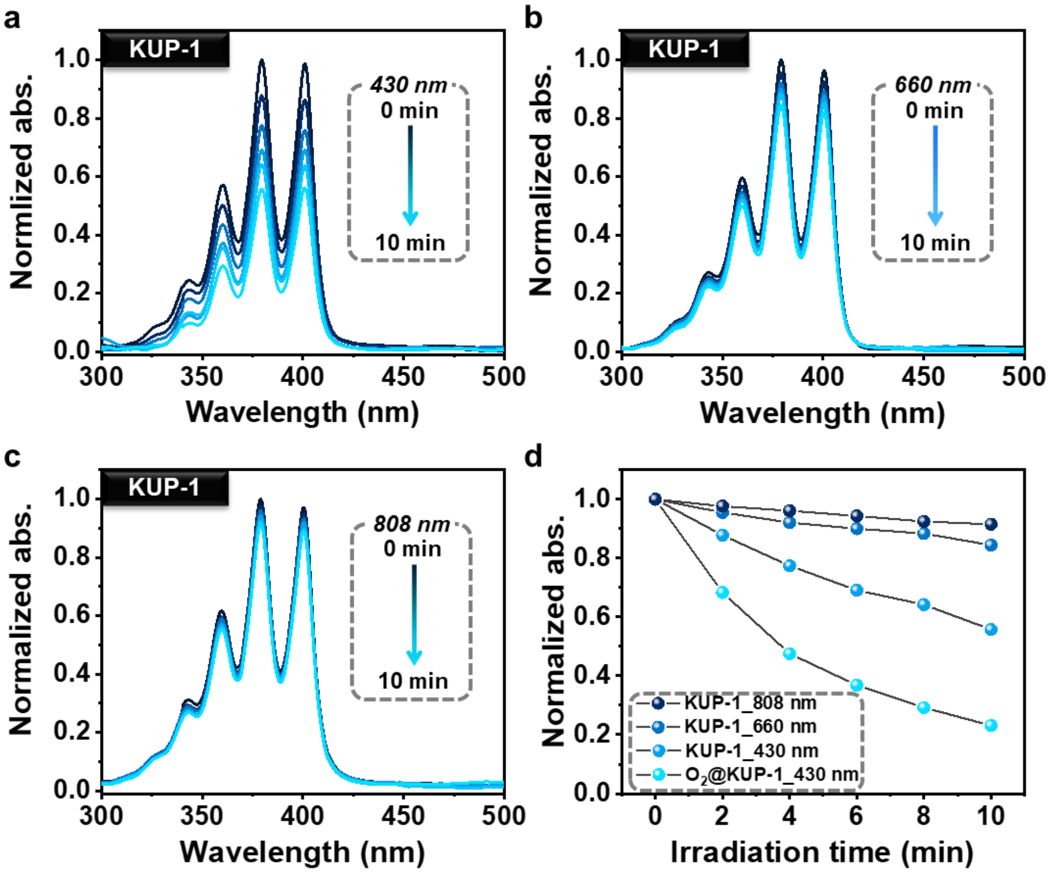

Supplementary Fig. 16 Evaluation on $^1O_2$ generation ability of **KUP-1** (0.2 mg mL^{-1}) using ABDA indicator with different wavelengths. UV-Vis absorbance spectra of ABDA (100 μM) in PBS solution were collected upon irradiation at each wavelength (**a**: 430, **b**: 660, and **c:** 808 nm) with a xenon lamp (1 mW cm^{-2}). **d** Normalized absorbance intensity comparison of Supplementary Figs. 16a, 16b, 16c, and Fig. 2b.

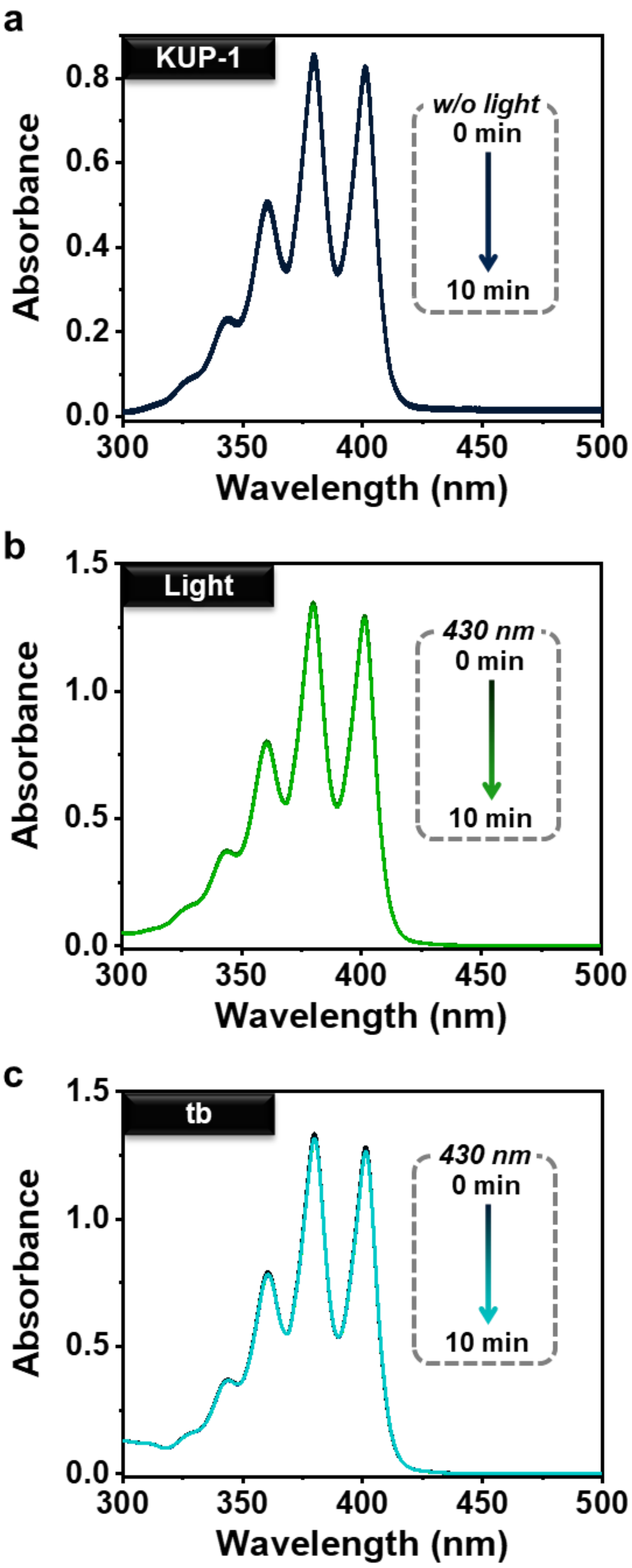

Supplementary Fig. 17 Evaluation on $^1O_2$ generation ability of control groups. **a** **KUP-1** (0.2 mg mL^{-1}, w/o irradiation), **b** light irradiation only without **KUP-1**, and **c** light irradiation only with 1,3,5-triformylbenzene (tb) (10 μM), respectively. UV-Vis absorbance spectra of ABDA (100 μM) in PBS solution were collected upon irradiation at 430 nm with a xenon lamp (1 mW cm^{-2}).

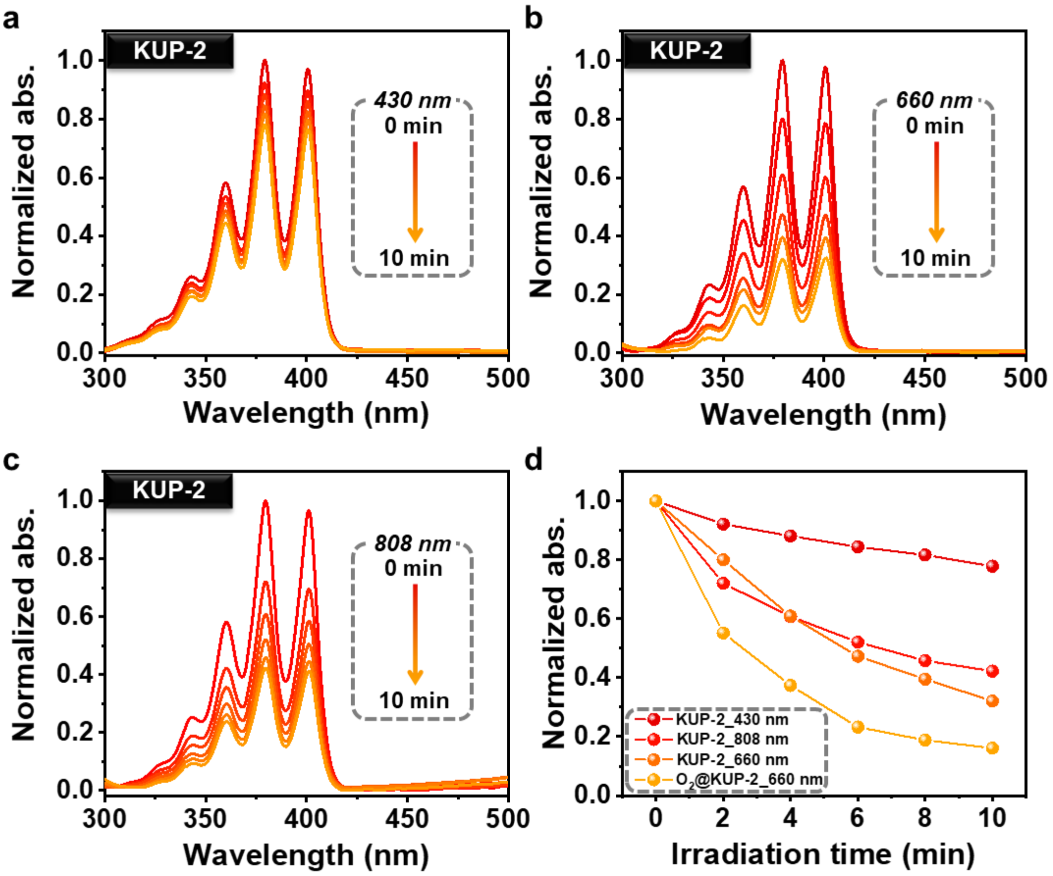

Supplementary Fig. 18 Evaluation on $^1O_2$ generation ability of **KUP-2** (0.2 mg mL^{-1}) using ABDA indicator with different wavelengths. UV-Vis absorbance spectra of ABDA (100 μM) in PBS solution were collected upon irradiation at each wavelength (**a**: 430, **b**: 660, and **c:** 808 nm) with a xenon lamp (1 mW cm^{-2}). **d** Normalized absorbance intensity comparison of Supplementary Figs. 18a, 18b, 18c, and Fig. 2c.

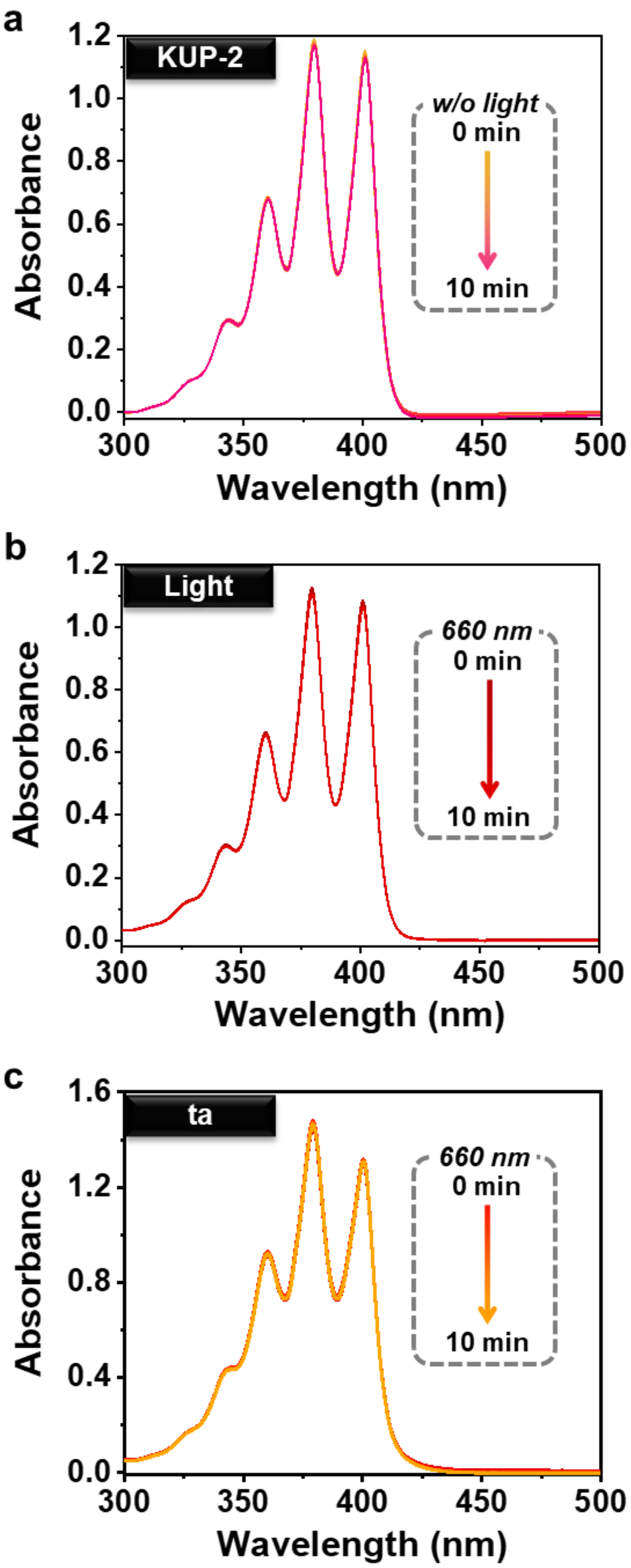

Supplementary Fig. 19 Evaluation on $^{1}O_2$ generation ability of control groups. **a** **KUP-2** (0.2 mg mL^{-1}, w/o irradiation), **b** light irradiation only without **KUP-2**, and **c** light irradiation only with tri(4-formylbenzene)amine (ta) (10 μM), respectively. UV-Vis absorbance spectra of ABDA (100 μM) in PBS solution were collected upon irradiation at 660 nm with a xenon lamp (1 mW cm^{-2}).

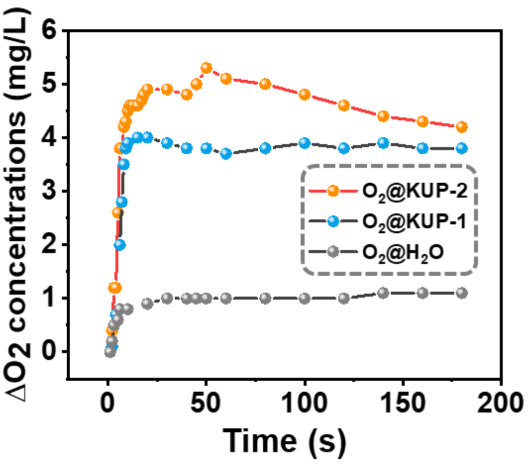

Supplementary Fig. 20 Dissolved oxygen curve of **O_2@KUP-1** and **O_2@KUP-2**. Compared with O_2@H_2O, dissolved oxygen capacity in the presence of each POP was significantly enhanced by its porosity and positive surface charge. Before the curve measurements, the baseline was corrected by degassed water.

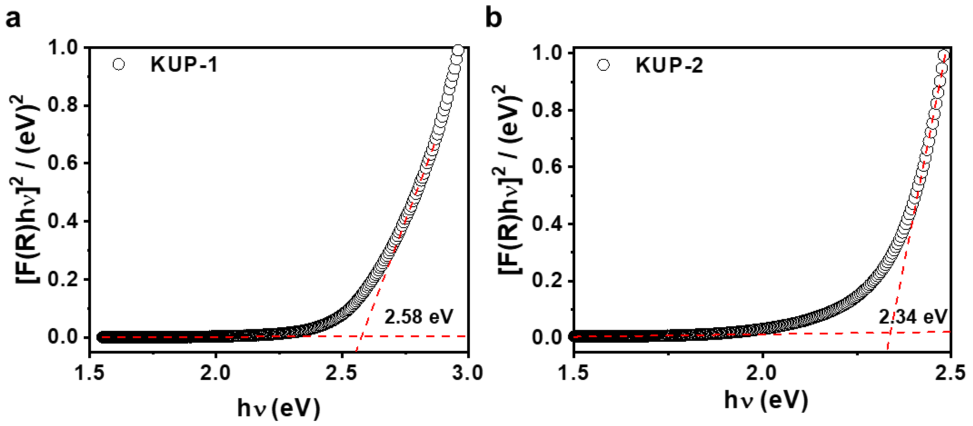

Supplementary Fig. 21 Tauc plots of POPs converted from Figure S15 to evaluate the band gap. The band gap was calculated to be **a** 2.58 for **KUP-1** and **b** 2.34 eV for **KUP-2** from fitted lines.

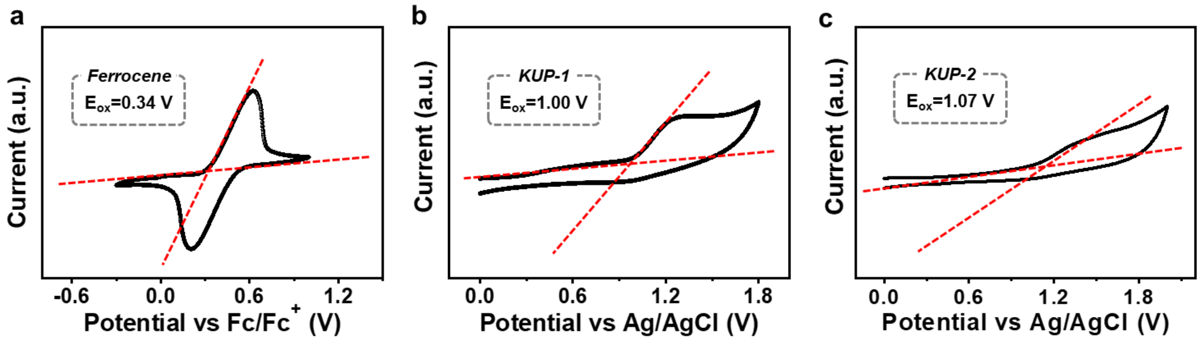

Supplementary Fig. 22 Cyclic voltammetry data of **a** ferrocene, **b KUP-1**, and **c KUP-2**. Before the measurement of the energy level of POPs, the experiment was conducted using ferrocene as a reference in anhydrous acetonitrile with tetrabutylammonium hexafluorophosphate (0.1 M). The working electrode was prepared by drop-casting a water/isopropyl alcohol suspension of POPs with 5 wt% Nafion.

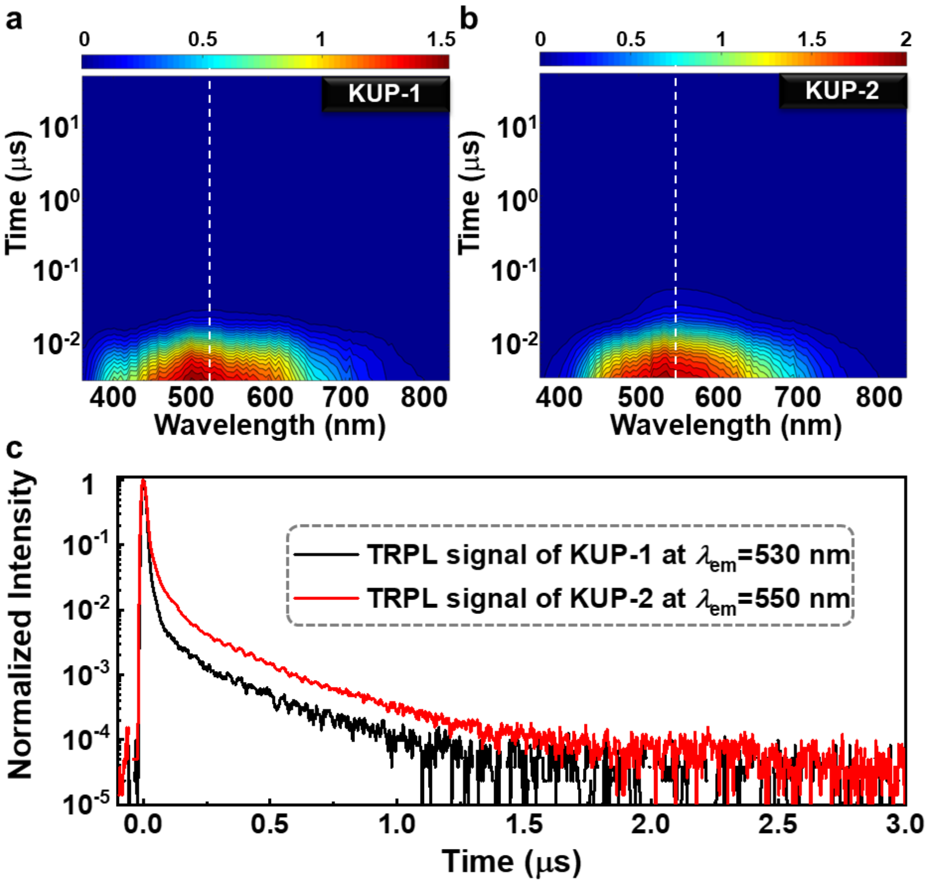

Supplementary Fig. 23 Wavelength-dependent time-resolved photoluminescence (TRPL) signals measured with **a KUP-1** and **b KUP-2** at 78 K. **c** TRPL signal of **KUP-1** and **KUP-2** at λ_{em} =530 and 550 nm, respectively.

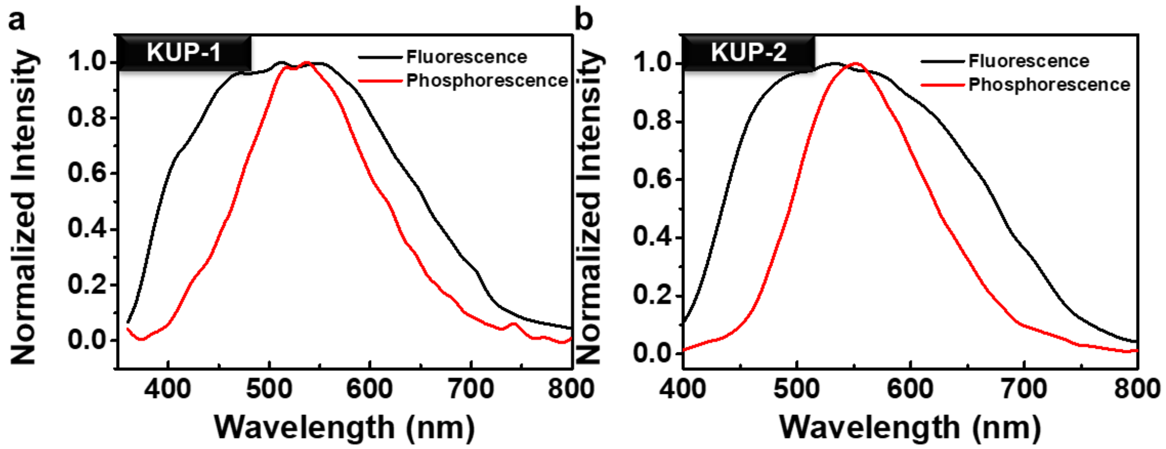

Supplementary Fig. 24 The fluorescence and phosphorescence spectra of **a KUP-1** and **b KUP-2** extracted from the wavelength-dependent TRPL signals.

Section IV. Mechanism-related studies of porous organic photosensitizers

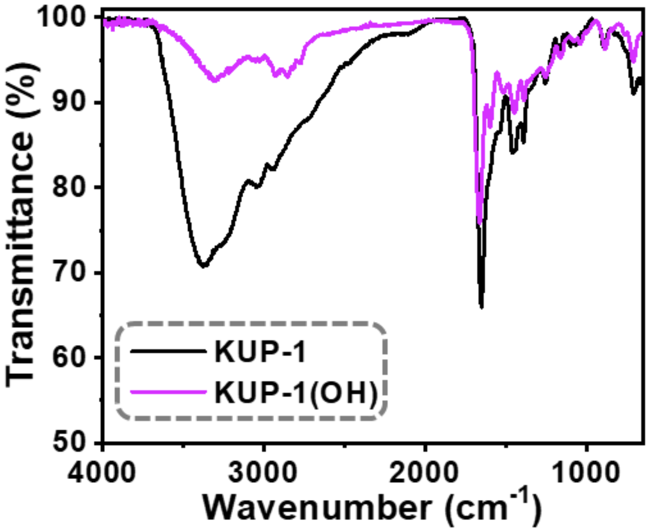

Supplementary Fig. 25 IR spectra of **KUP-1** and **KUP-1(OH)**. After hydroxide ion treatment, the peaks were noticeably changed in the range of 2500-3600 cm^{-1}.

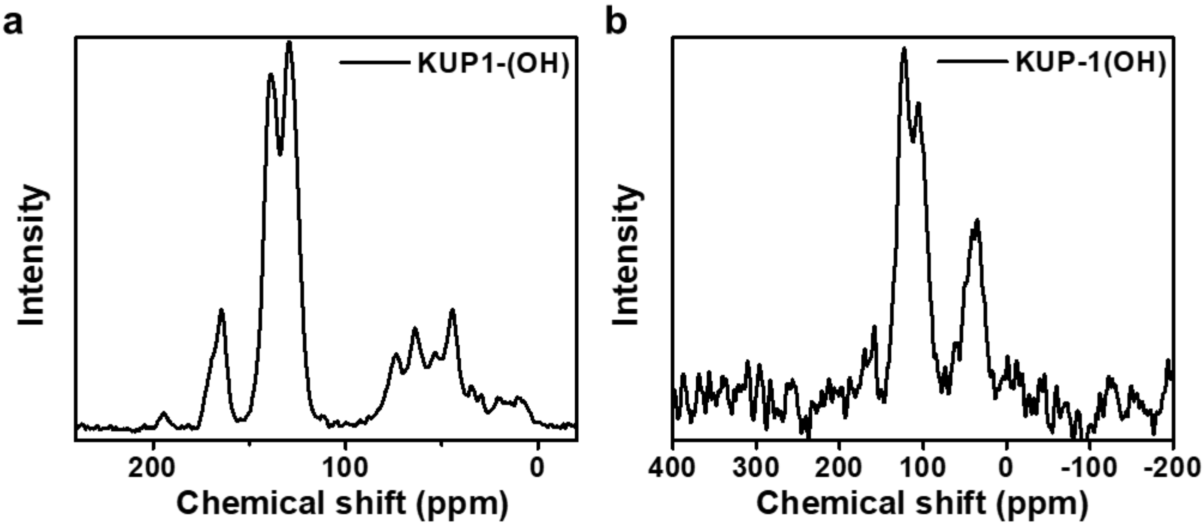

Supplementary Fig. 26 Solid-state **a** ^{13}C NMR and **b** ^{15}N data of **KUP-1(OH)**. The chemical environment of **KUP-1** was changed after hydroxide ion treatment.

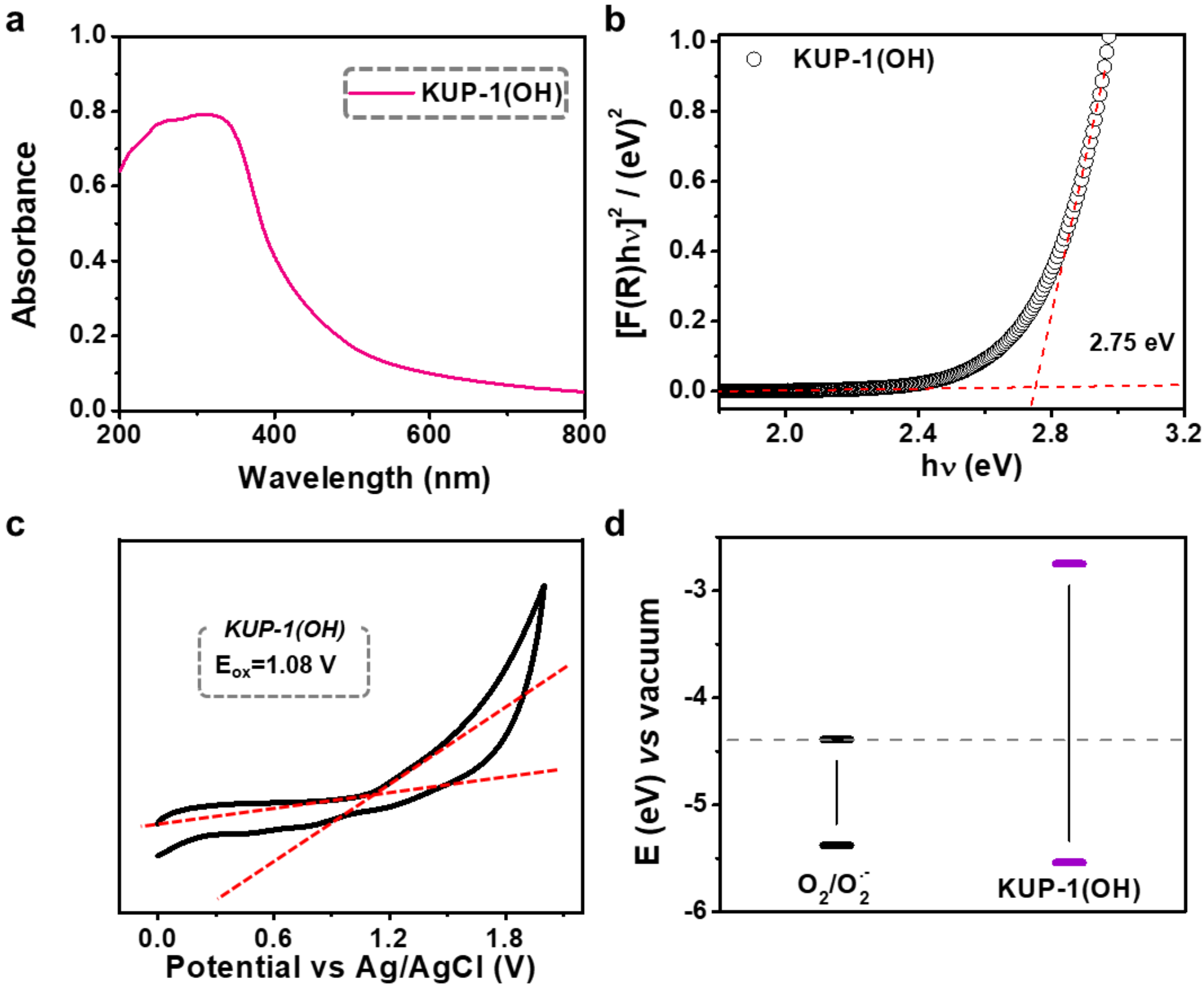

Supplementary Fig. 27 a Solid-state UV-Vis spectrum of **KUP-1(OH)**. **b** Tauc plot of **KUP-1(OH)**. From the plot, the band gap of **KUP-1(OH)** was calculated to be 2.75 eV. **c** Cyclic voltammetry oxidative data of **KUP-1(OH)**. The oxidation data were measured in the same experimental condition as in Supplementary Fig. 21. **d** Band gap diagram of **KUP-1(OH)**.

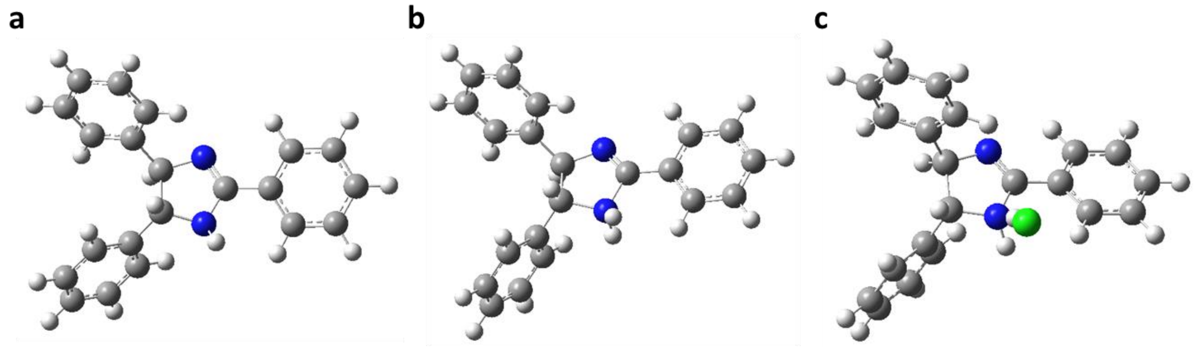

Supplementary Fig. 28 Optimized structures of **a KUP-1(OH)** monomer. **b** model **KUP-1** (**KUP-1$_m$**) monomer. **c KUP-1** monomer.

Supplementary Table 1 Calculated spin-orbit coupling constant ($\langle \boldsymbol{S_1}|\boldsymbol{H_{SOC}}|\boldsymbol{T_1}\rangle$), the energy difference between the S1 and T1 states (DEST), reorganization energy (l), and minimum energy crossing point (MECP) of **KUP-1(OH)**, **KUP-1$_\mathbf{m}$**, and **KUP-1** systems.

	$\langle S_1|H_{SOC}|T_1\rangle$ (cm^{-1})		ΔE_{st} (eV)		λ (eV)		MECP (eV)	
	Mono.	Olig.	Mono.	Olig.	Mono.	Olig.	Mono.	Olig.
**KUP-1(OH)**	1.73	1.68	1.82	2.55	2.60	3.73	5.83×10^{-2}	9.29×10^{-2}
**KUP-1$_\mathbf{m}$**	0.27	0.08	0.50	0.47	2.11	0.39	3.06×10^{-1}	3.86×10^{-3}
**KUP-1**	12.80	-	0.44	-	0.40		6.19×10^{-4}	

Supplementary Table 2 Computational absorption energies per oxygen molecule on **KUP-1(OH)** and **KUP-1$_\mathbf{m}$** according to the number of oxygen molecules.

kcal/mol		Number of oxygen molecule		
		n = 1	n = 2	n = 3
**KUP-1$_\mathbf{m}$**	Oligomer	11.21	13.56	17.28
**KUP-1(OH)**	Oligomer	1.57	-1.79	1.48

Section V. Biological data of porous organic photosensitizers

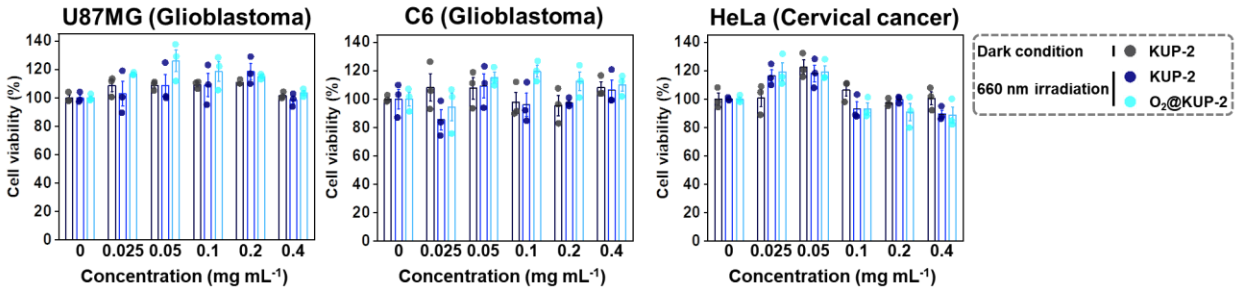

Supplementary Fig. 29 The cytotoxicity ability assay of **KUP-2** and **O_2@KUP-2** after irradiation at 660 nm. The U87MG, C6, and HeLa cell lines were used. The incubation of **KUP-2** and **O_2@KUP-2** was fixed at 12 h for being uptaken into the cytosol. The measurement of toxicity was performed after incubation for 24 h. The error bar represents mean ± S.E.M. (n=3). The dot plots represent Jitter points.

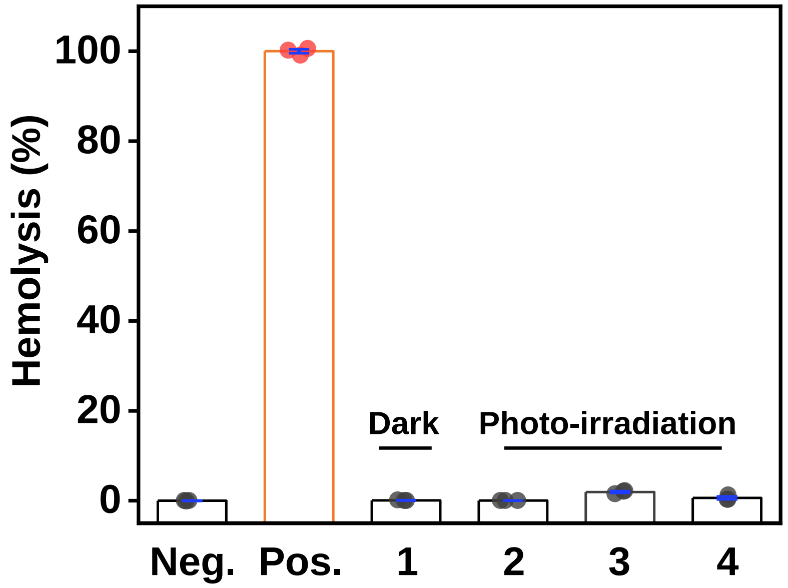

Supplementary Fig. 30 Hemolysis test for **KUP-2** (0.4 mg mL^{-1}) and **O_2@KUP-2** (0.4 mg mL^{-1}). Neg: negative control (PBS), Pos: positive control; 0.1% (v/v) Triton X-100. 1: the group which KUP-2 treats without irradiation, 2: the group, which is treated by PBS with irradiation, 3: the group which is treated by **KUP-2** with irradiation4: the group which is treated by **O_2@KUP-2** with irradiation. An inset photograph is a supernatant of the damaged red blood cells. The error bar represents mean ± S.E.M. (n=3); each column has ± 0.04519% (Neg), ± 0.44506% (Pos), ± 0.045187% (Dark, Treat), ± 0.207075 (Light, non-treat), and ± 0.316313% (Light, treat) respectively. The dot plots represent Jitter points.

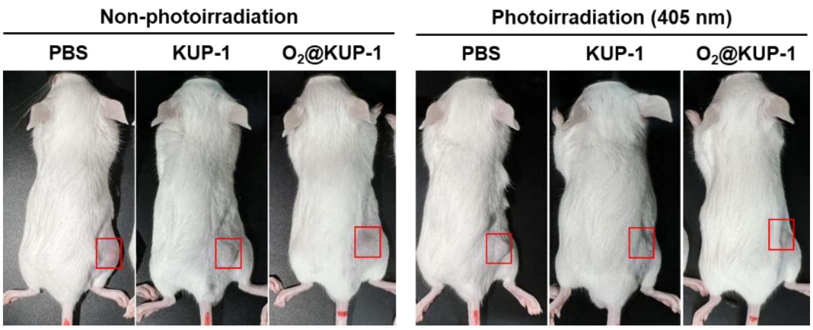

Supplementary Fig. 31 Images of mouse condition after treatment of 1×PBS, **KUP-1** (8 mpk; mg/kg), and **O_2@KUP-1** (8 mpk) with/without photo-induced treatment for 13 days.

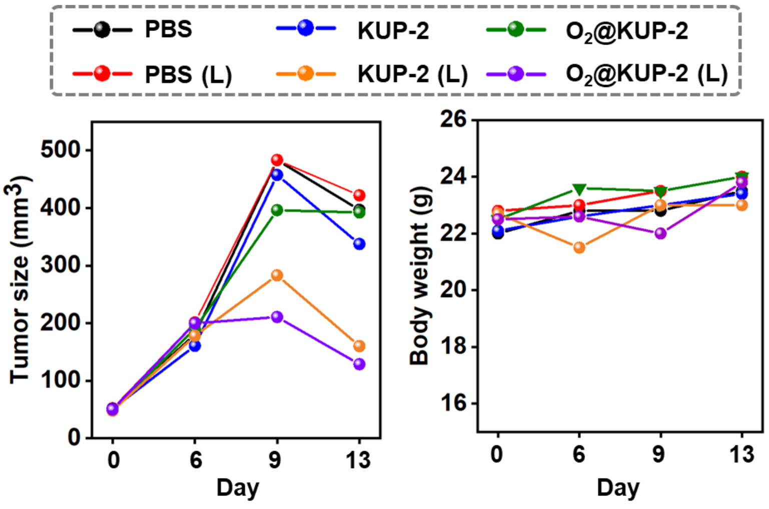

Supplementary Fig. 32 Tumor sizes and body weights of the mice in each tested group recorded during treatment with/without 660 nm irradiation at the end-point (n = 5, number of mice). (L): laser.
