# Supplementary material for: Wavelength engineerable porous organic polymer photosensitizers with protonation triggered ROS generation
Source: Nat Commun. 2023 Mar 17;14:1498. doi: 10.1038/s41467-023-37156-x (PMC10023675; doi:10.1038/s41467-023-37156-x)
Supplement: Supplementary file 3 — Reporting Summary [file 41467_2023_37156_MOESM3_ESM.pdf]

nature portfolio

Corresponding author(s): Chang Seop Hong

Last updated by author(s): Mar 2, 2023

Reporting Summary

Nature Portfolio wishes to improve the reproducibility of the work that we publish. This form provides structure for consistency and transparency in reporting. For further information on Nature Portfolio policies, see our Editorial Policies and the Editorial Policy Checklist.

Statistics

For all statistical analyses, confirm that the following items are present in the figure legend, table legend, main text, or Methods section.

n/a	Confirmed	
☐	☒	The exact sample size (*n*) for each experimental group/condition, given as a discrete number and unit of measurement
☐	☒	A statement on whether measurements were taken from distinct samples or whether the same sample was measured repeatedly
☐	☒	The statistical test(s) used AND whether they are one- or two-sided<br>*Only common tests should be described solely by name; describe more complex techniques in the Methods section.*
☐	☒	A description of all covariates tested
☐	☒	A description of any assumptions or corrections, such as tests of normality and adjustment for multiple comparisons
☐	☒	A full description of the statistical parameters including central tendency (e.g. means) or other basic estimates (e.g. regression coefficient) AND variation (e.g. standard deviation) or associated estimates of uncertainty (e.g. confidence intervals)
☐	☒	For null hypothesis testing, the test statistic (e.g. F, t, r) with confidence intervals, effect sizes, degrees of freedom and P value noted<br>*Give P values as exact values whenever suitable.*
☒	☐	For Bayesian analysis, information on the choice of priors and Markov chain Monte Carlo settings
☒	☐	For hierarchical and complex designs, identification of the appropriate level for tests and full reporting of outcomes
☒	☐	Estimates of effect sizes (e.g. Cohen's *d*, Pearson's *r*), indicating how they were calculated

Our web collection on statistics for biologists contains articles on many of the points above.

Software and code

Policy information about availability of computer code

Data collection	ZEISS ZEN 2 (blue edition, Oberkochen, Germany), VISQUE CleVue 1 (Vieworks, Anyang, Rep. of Korea), VASP 5.3.5, Qchem 5.3.2, PYXAID 1.0, Gaussian16 Revision A.03
Data analysis	ZEISS ZEN 2 (blue edition, Oberkochen, Germany), GraphPad InStat version 8.0.1 (GraphPad Software, La Jolla, CA, USA), VISQUE CleVue 1 (Vieworks, Anyang, Rep. of Korea), VASP 5.3.5, Qchem 5.3.2, PYXAID 1.0, Gaussian16 Revision A.03

For manuscripts utilizing custom algorithms or software that are central to the research but not yet described in published literature, software must be made available to editors and reviewers. We strongly encourage code deposition in a community repository (e.g. GitHub). See the Nature Portfolio guidelines for submitting code & software for further information.

Data

Policy information about availability of data

All manuscripts must include a data availability statement. This statement should provide the following information, where applicable:

- Accession codes, unique identifiers, or web links for publicly available datasets
- A description of any restrictions on data availability
- For clinical datasets or third party data, please ensure that the statement adheres to our policy

The authors declare that the data supporting the findings of this study are available within the Article and its Supplementary Information or from the corresponding author upon reasonable request.

Human research participants

Policy information about studies involving human research participants and Sex and Gender in Research.

Reporting on sex and gender	N/A
Population characteristics	N/A
Recruitment	N/A
Ethics oversight	N/A

Note that full information on the approval of the study protocol must also be provided in the manuscript.

Field-specific reporting

Please select the one below that is the best fit for your research. If you are not sure, read the appropriate sections before making your selection.

☒ Life sciences ☐ Behavioural & social sciences ☐ Ecological, evolutionary & environmental sciences

For a reference copy of the document with all sections, see nature.com/documents/nr-reporting-summary-flat.pdf

Life sciences study design

All studies must disclose on these points even when the disclosure is negative.

Sample size	No sample-size calculation was performed. The each sample sizes were chosen as five number every group. Although we did not set up the sample size using the sample-size calculation, the data were obtained as well-reproductive results even in the independent condition (experiment data; interval: about 1 month).
Data exclusions	No data were excluded from the analyses.
Replication	All attempts at replication were successful. We independently performed the three time experiment in other data (Interval: about 1 month).
Randomization	We allocated randomly the in vitro/vivo samples that were planed to be treated.
Blinding	It was not possible to analyze the results using the blinding test. First, we must mark the group name, count of treatment, and experiment date on the cage because we have been thoroughly monitored under Ethics Council of University. Second, we need to check the information which group is treated by our material before photo-irradiation. We alternatively performed the reproductive analysis even though we did not consider the blind test.

Reporting for specific materials, systems and methods

We require information from authors about some types of materials, experimental systems and methods used in many studies. Here, indicate whether each material, system or method listed is relevant to your study. If you are not sure if a list item applies to your research, read the appropriate section before selecting a response.

Materials & experimental systems

n/a	Involved in the study	
☐	☒	Antibodies
☐	☒	Eukaryotic cell lines
☒	☐	Palaeontology and archaeology
☐	☒	Animals and other organisms
☒	☐	Clinical data
☒	☐	Dual use research of concern

Methods

n/a	Involved in the study	
☒	☐	ChIP-seq
☒	☐	Flow cytometry
☒	☐	MRI-based neuroimaging

Antibodies

Antibodies used	We have used the Proteome Profiler Array Kit (Catalog Number: ARY006) based on principle of the antigen-antibody. The mixture of capture antibody is consist of next list; CXCL13/BCA-1, C5/C5a, G-CSF, GM-CSF, I-309, Eotaxin, ICAM-1, IFN-γ, IL-1α, IL-1β, IL-1ra, IL-2, IL-3, IL-4, IL-5, IL-6, IL-7, IL-10, IL-13, IL-12p70, IL-16, IL-17, IL-23, IL-27.

Validation	The Proteome Profiler Array Kit (Catalog Number: ARY006) was provided from R&D systems (US, http://rndsystems.com)

Eukaryotic cell lines

Policy information about cell lines and Sex and Gender in Research

Cell line source(s)	U87MG cell was obtained from the Korean Cell Line Bank (KCLB)
Authentication	We obtained the KCLB No. 30014 cell line (U87MG) that is certified by the Korean Cell Line Bank (KCLB) without additional authentication service
Mycoplasma contamination	We declare that the cell lines were not tested for mycoplasma contamination.
Commonly misidentified lines (See ICLAC register)	We did not use the misidentified cell lines in this work.

Animals and other research organisms

Policy information about studies involving animals; ARRIVE guidelines recommended for reporting animal research, and Sex and Gender in Research

Laboratory animals	Five-week-old male, BALB/c
Wild animals	This study did not involve the wild animals
Reporting on sex	This study applied to only one sex (male). We think the sex-based analysis is not required in our experiment because this study was not experiment related to hormone of each male and female
Field-collected samples	This study did not involve samples collected from the field
Ethics oversight	The experiments performed with mice were carried out in accordance with the National Institute of Health Guide for the Care and Use of Laboratory Animals (NIH publications no. 80-23, revised 1996) and protocols approved by the Institutional Animal Care and Use Committee of Kyung Hee University for each experiment (KHUASP- (SE)-19-002 and KHUASP(SE)-19-003) and Institutional guidelines (assigned no. 2015-020)

Note that full information on the approval of the study protocol must also be provided in the manuscript.
